# Epstein-Barr Virus-Positive Mucocutaneous Ulcer on the Gingiva of a Patient in Whom Immunosuppressive Drugs Could Not Be Withdrawn: A Case Report and Review of the Literature

**DOI:** 10.7759/cureus.56176

**Published:** 2024-03-14

**Authors:** Tatsuya Sakaguchi, Shunichi Yoshida, Takeshi Karube, Taneaki Nakagawa, Seiji Asoda

**Affiliations:** 1 Department of Dentistry and Oral Surgery, Keio University School of Medicine, Tokyo, JPN; 2 Department of Dentistry and Oral Surgery, National Hospital Organization, Kasumigaura Medical Center, Ibaraki, JPN

**Keywords:** lymphoproliferative disorder, mucocutaneous ulcer, oral cavity environment, methotrexate, ebv-positive mucocutaneous ulcer

## Abstract

Epstein-Barr virus-positive mucocutaneous ulcer (EBV-MCU) is characterized by ulcers confined to the skin and mucus membranes. EBV-MCU is an EBV-positive lymphoproliferative disorder that occurs during the use of immunosuppressive drugs such as methotrexate. We herein report a case of EBV-MCU in the maxillary gingiva. A 73-year-old woman was referred to our department in March 2021. During the initial examination, bone exposure and ulceration were observed in the extraction socket of the maxillary bilateral central incisors. The patient was taking methotrexate for rheumatoid arthritis and was unable to stop due to disease progression. In March 2021, curettage of the extraction socket of the maxillary anterior teeth and extraction of the maxillary right lateral incisor, which was difficult to preserve due to severe tooth mobility, was performed under local anesthesia. The extraction site epithelialized and healed well. Three months later, inflammation flared, and ulceration was observed. Extraction of the unsalvageable maxillary teeth and an excisional biopsy of the palatal gingiva were performed. The histopathological diagnosis was EBV-MCU. The postoperative course was uneventful, and no evidence of recurrence was found two years postoperatively; follow-up will be continued. There are many reports of EBV-MCU remission with the cessation of methotrexate treatment. In our patient, withdrawal was difficult because of the progression of rheumatoid arthritis, but remission was achieved by improving the oral cavity environment through an excisional biopsy and tooth extraction.

## Introduction

Epstein-Barr virus-positive mucocutaneous ulcer (EBV-MCU) is a new categorization of EBV-positive lymphoproliferative disorder (EBV-LPD) among mature B-cell tumors, according to the 2017 WHO classification [1]. EBV-MCU is characterized by localized ulceration of the skin, oral mucosa, pharyngeal mucosa, and gastrointestinal tract. The oral mucosa is chronically irritated by oral bacteria, dental caries, periodontal disease, poor prosthetics, and bites, and EBV-MCU is thought to have a predilection for the oral mucosa. They are broadly classified into iatrogenic and age-associated types. Iatrogenic cases occur with the use of immunosuppressive drugs such as methotrexate (MTX), prednisolone (PSL), or other immunosuppressive conditions. EBV-MCU has a better prognosis than other EBV-LPDs, and it often remits after the withdrawal of immunosuppressive drugs.

We herein report a case of EBV-MCU in the maxillary gingiva in which MTX was difficult to withdraw, but remission was achieved with an improvement of the local environment.

## Case presentation

**Figure 1:**
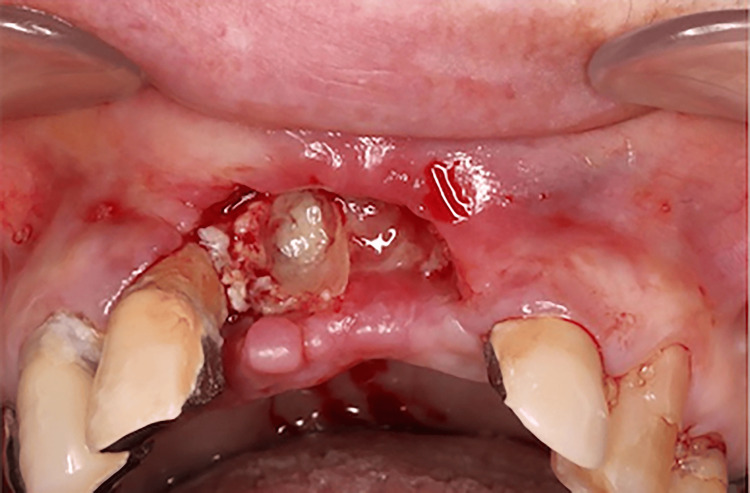
Intraoral photographs at the initial examination Bone exposure of the extraction socket of bilateral maxillary central incisors was observed.

**Figure 2:**
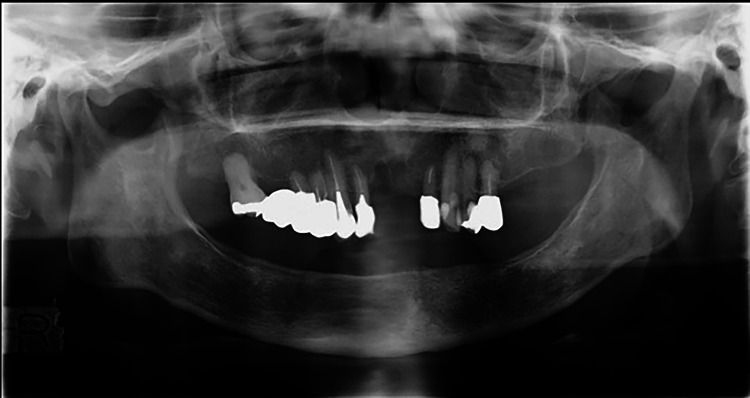
Panoramic radiograph. A remnant extraction socket of the right maxillary central incisor and hardening of the alveolar bone line were observed.

**Figure 3:**
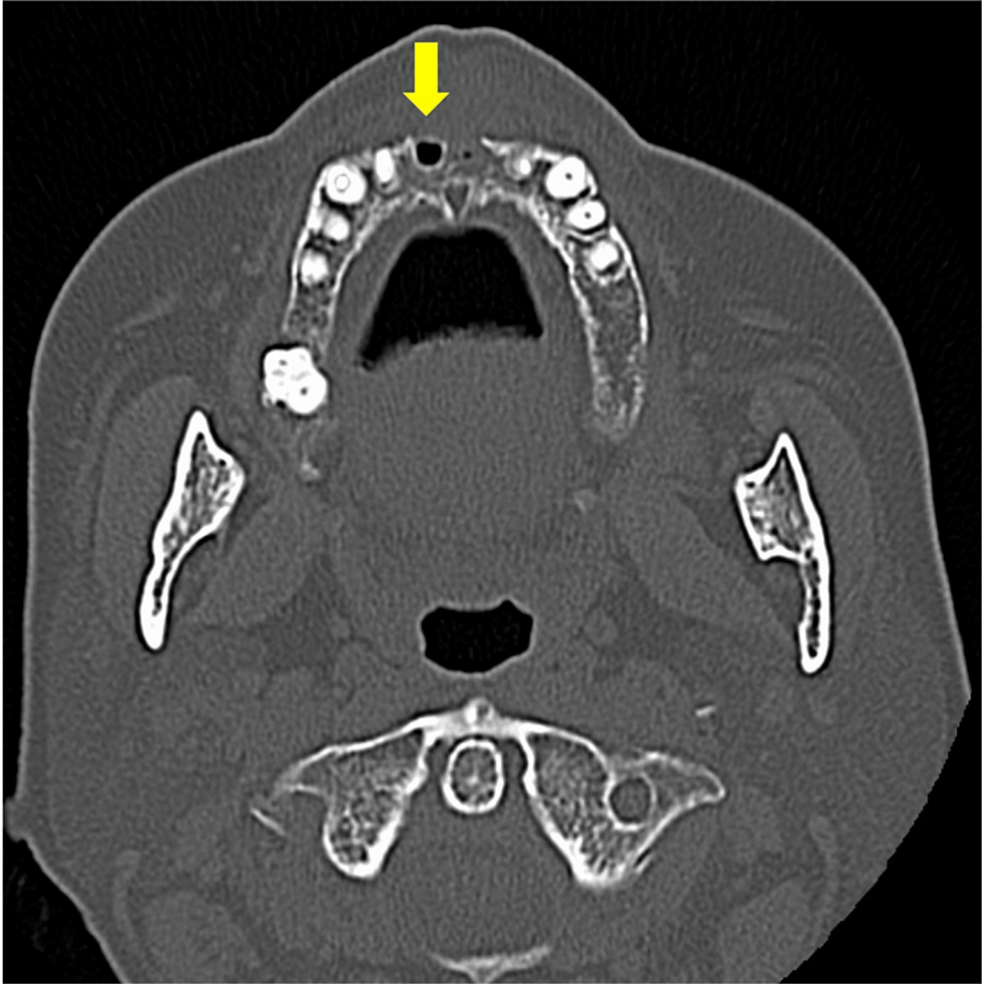
CBCT findings There was no evidence of sequestration or bone destruction, and cortical bone-like osteosclerosis (arrow) was seen around the extraction socket of the right maxillary central incisor.

In March 2021, a 73-year-old woman was referred to our department for a thorough examination due to failure of healing after extraction of the maxillary bilateral central incisors at her family dentist's office. The patient had a history of progressive rheumatoid arthritis, had been taking MTX for a long time, and had a thin build. Her other medical history was hypercholesterolemia. There was no family history or allergies. Medications in use were Foliamin 5 mg/week, rosuvastatin calcium 2.5 mg/day, methotrexate 10 mg/week, and Iguratimod 25 mg/day. She had deformities of the hand and ankle joints due to rheumatoid arthritis. No skin lesions were observed. An intraoral examination revealed bone exposure in the extraction socket of the bilateral maxillary central incisors (Figure 1). The patient's oral hygiene was poor (plaque control record is over 90%), with many mobile teeth and the average depth of periodontal pockets was about 6 mm. Panoramic radiographs showed a residual extraction socket of the right maxillary central incisor and sclerosis of the alveolar hard line (Figure 2). Dental cone beam computed tomography (CBCT) revealed no apparent sequestration or bone destruction, and cortical bone-like sclerosis was observed around the extraction socket of the right maxillary central incisor (Figure 3). Based on these findings, we made a clinical diagnosis of healing failure of the extraction socket of the maxillary anterior tooth.

**Figure 4:**
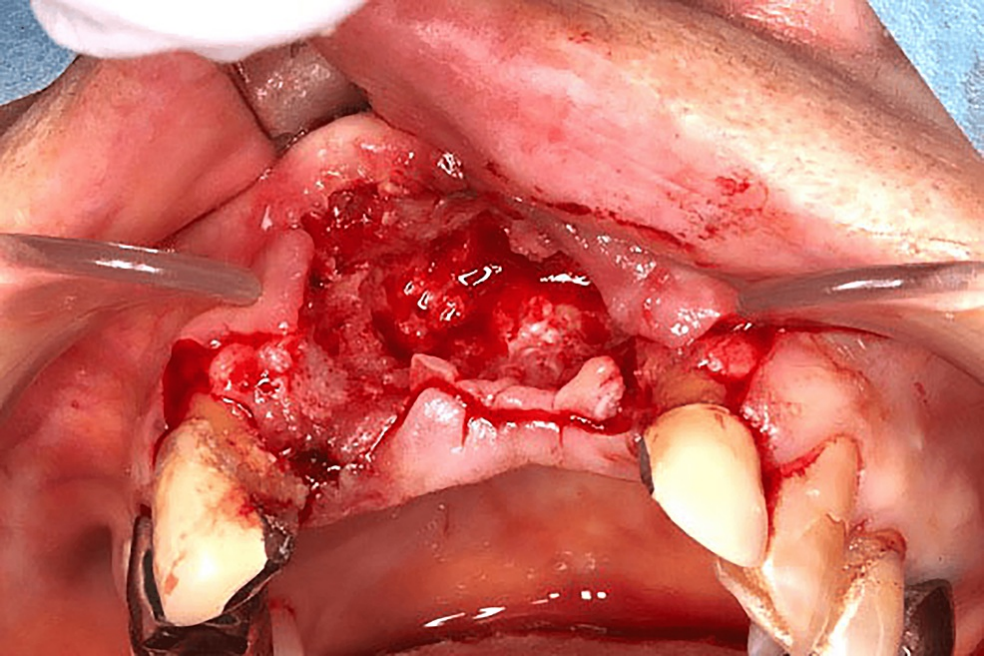
Initial treatment findings Bleeding from the bone was observed during the curettage of the extraction socket.

**Figure 5:**
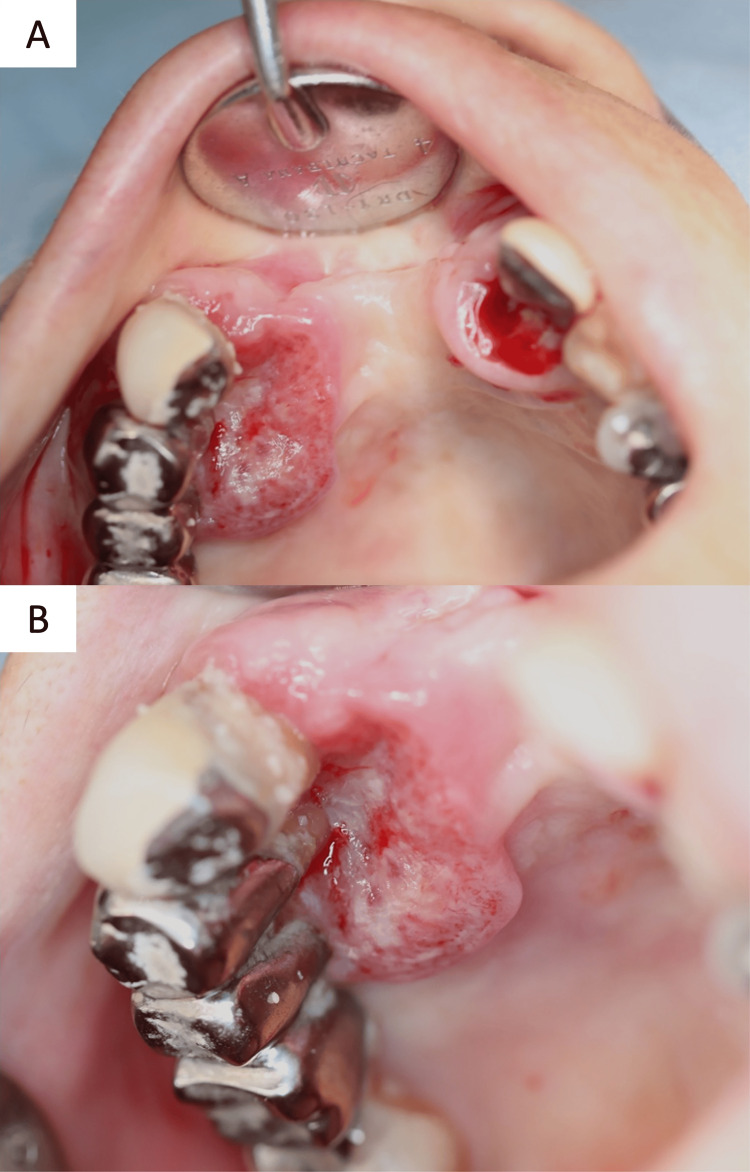
Intraoral findings at the time of the excisional biopsy A: Three months after the procedure, there was a flare-up of inflammation and ulceration with a mass. B: An enhanced image revealed that especially in the part of the gingiva of the right maxillary canine and premolar.

**Figure 6:**
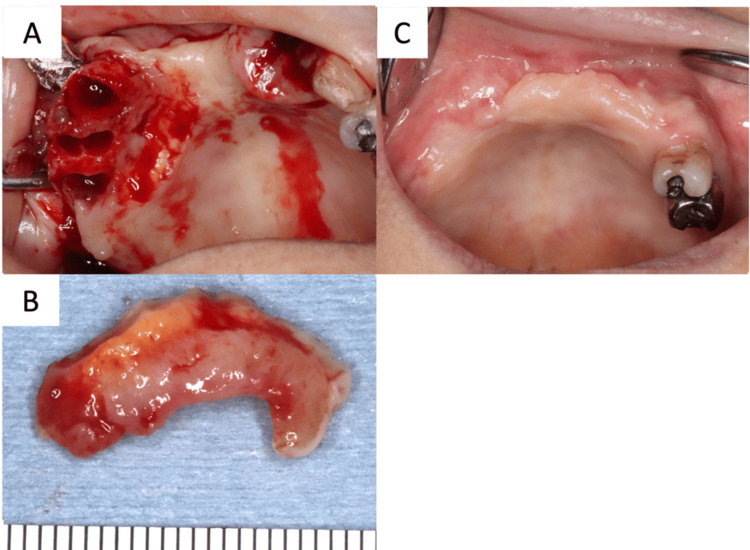
Intraoperative findings at the time of the excisional biopsy and intraoral findings after two years of follow-up A: Extraction of the right maxillary canine, first premolar, second premolar, second molar, the left maxillary lateral incisor, and canine, as well as an excisional biopsy of the palatal gingiva around the right maxillary canine and first premolar were performed. B: Resected lesion macrograph. C: Epithelialization is good and no recurrence is observed.

In March 2021, under local anesthesia, extraction socket curettage was performed on the maxillary anterior teeth and the right maxillary lateral incisor, which was extremely mobile and difficult to preserve, was extracted (Figure 4). Three months after the procedure, ulceration with a mass was observed around the palatal gingiva of the right maxillary canine, first premolar, second premolar, and second molar. Significant tooth mobility of the right maxillary canine, first premolar, second premolar, second molar, left maxillary lateral incisor, and canine was observed (Figure 5). The patient was taking MTX for rheumatoid arthritis and was suspected of having methotrexate-related lymphoproliferative disorder (MTX-LPD). The patient had advanced rheumatoid arthritis and was unable to discontinue MTX therapy. Therefore, local control treatment was planned. In June 2021, under local anesthesia, extraction of the right maxillary canine, first premolar, second premolar, second molar, and left maxillary lateral incisor, and canine along with an excisional biopsy, including the periosteum on the palatal and maxillary gingiva around the right maxillary canine and first premolar, were performed (Figures 6A, 6B).

**Figure 7:**
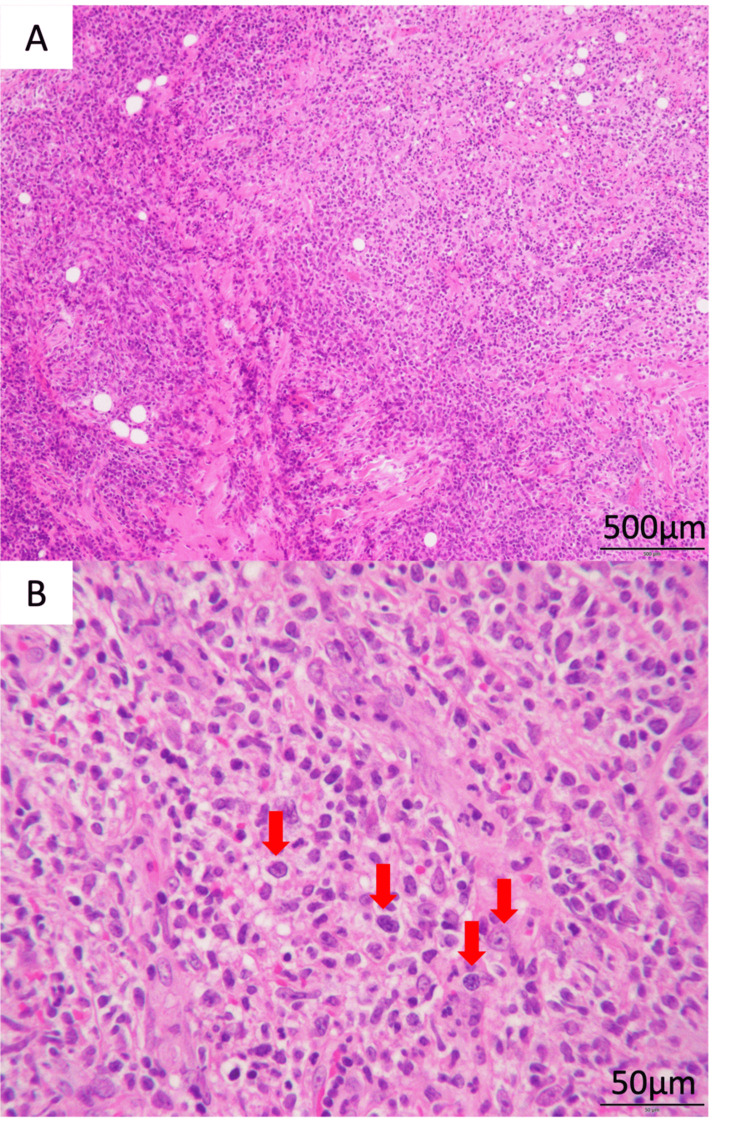
Histopathological findings (hematoxylin-eosin stain) The patient showed granulation and severe inflammation, and atypical cells were observed, resembling diffuse large-cell lymphoma (arrow). A: low magnification (×4); B: high magnification (×40)

**Figure 8:**
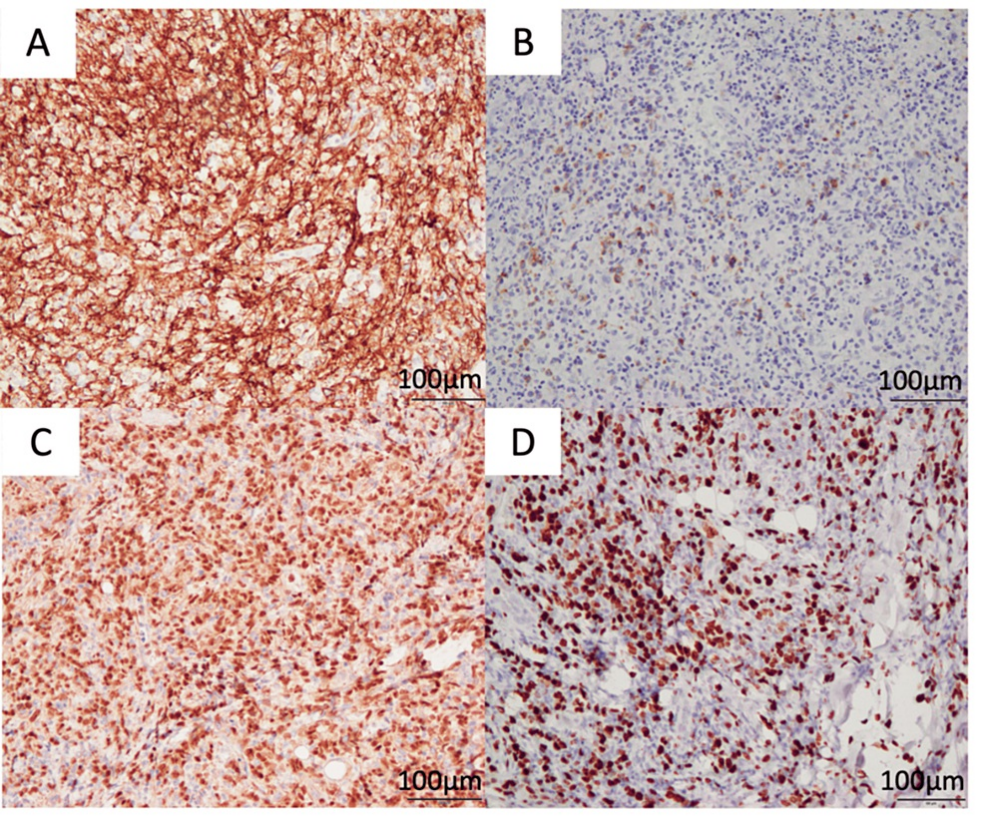
Immunohistochemical findings were similar to diffuse large-cell lymphoma (A) CD20 (×20); (B) LMP-1 (×20); (C) PAX-5 (×20); (D) Ki-67 (×20)

Histopathological findings showed granulation and high inflammation in the submucosal tissue with atypical cells on hematoxylin-eosin (HE) staining. The atypical cells had bare nuclei and irregularly shaped, distinct nucleoli (Figures 7A, 7B). Immunohistochemical staining was positive for CD20, LMP-1, and PAX5, and the Ki-67 index was positive in 80% of specimens (Figures 8A-8D). The histological picture resembled diffuse large-cell lymphoma, but the diagnosis of EBV-MCU was based on the history of MTX use for rheumatoid arthritis. The patient has not experienced any recurrence in the two years since surgery and is currently doing well. Follow-up will continue (Figure 6C).

## Discussion

EBV-MCU is an LPD classified as a mature B-cell tumor and was first reported by Dojcinov et al. in 2010 [2] before being newly established in the 2017 WHO classification. It is reportedly more common in women than men and is present in the mucosa of the oropharynx (52%), skin (29%), or gastrointestinal tract (19%) [3]. Seventy-eight cases of EBV-MCU occurring in the oral cavity have been reported since 2017, when the WHO classification of EBV-MCU was established, including our own cases (Table 1) [4-24]. The mean age of the cases was 71.6 years old, and the male-to-female ratio was 32:47, similar to that in previous reports. The most common sites were the maxillary and mandibular gingiva, palate, tongue, and mucosa of the lips.

**Table 1:** Previous reports of EBV-MCU in the oral cavity D, dead; CR, complete remission; PR, partial remission; N.D., no data; +, positive; CLL, chronic lymphocytic leukemia; IS, immunosuppression; RA, rheumatoid arthritis; DH, dermatitis herpetiformis; DLBCL, diffuse large B-cell; MTX, methotrexate; LMF, leflunomide; PSL, prednisolone; MPA, mycophenolic acid; TAC, tacrolimus; BUC, bucillamine; HU, hydroxycarbamide; AZA, azathioprine; SASP, salazosulfapyridine lymphoma; RIX, rituximab; R-THPCOP, rituximab, cyclophosphamide, pirarubicin, vincristine, prednisolone; R-COEP, rituximab, cyclophosphamide, oncovin, etoposide, prednisolone; R-CHOP, rituximab, cyclophosphamide, hydroxydaunorubicin, oncovin, prednisolone; FCR, fludarabine, cyclophosphamide, rituximab; ABVD, doxorubicin, bleomycin, vinblastine, dacarbazine

							immunohistochemical features				
Study	Sex	Age, years	Location	Source of immunosuppression	Response	Management	LMP-1	EBER	CD20	Ki67	PAX5
Aldridge et al. 2017 [21]	M	83	Gingiva	IS (MTX for RA therapy)	CR	Reduce IS	N.D.	N.D.	＋	N.D.	N.D.
Natkunam et al.2017 [22]	F	49	Oral mucosa	N.D.	recurrence	N.D.	N.D.	N.D.	N.D.	N.D.	N.D.
	F	77	Palate	N.D.	N.D.	N.D.	N.D.	N.D.	N.D.	N.D.	N.D.
	M	77	Oral mucosa	N.D.	CR	N.D.	N.D.	N.D.	N.D.	N.D.	N.D.
	F	49	Oral mucosa	N.D.	CR	N.D.	N.D.	N.D.	N.D.	N.D.	N.D.
	F	17	Oral mucosa	N.D.	N.D.	N.D.	N.D.	N.D.	N.D.	N.D.	N.D.
	M	81	Oral mucosa	N.D.	N.D.	N.D.	N.D.	N.D.	N.D.	N.D.	N.D.
	F	63	Lip	N.D.	N.D.	N.D.	N.D.	N.D.	N.D.	N.D.	N.D.
Ohata et al. 2017 [23]	M	81	Gingiva	Age	CR	R-COEP	N.D.	+	N.D.	N.D.	N.D.
	M	69	Gingiva	Age	CR	R-CHOP	N.D.	+	N.D.	N.D.	N.D.
	M	83	Gingiva	Age	N.D.	R-CHOP	N.D.	+	N.D.	N.D.	N.D.
	M	79	Gingiva	Age	CR	R-CHOP	N.D.	+	N.D.	N.D.	N.D.
	F	84	Gingiva	N.D.	CR	Reduce IS	N.D.	+	N.D.	N.D.	N.D.
	F	81	Gingiva	N.D.	N.D.	Reduce IS	N.D.	+	N.D.	N.D.	N.D.
	F	58	Gingiva	N.D.	CR	Reduce IS	N.D.	+	N.D.	N.D.	N.D.
	F	71	Gingiva	N.D.	CR	Reduce IS	N.D.	+	N.D.	N.D.	N.D.
	M	70	Gingiva	N.D.	CR	Reduce IS	N.D.	+	N.D.	N.D.	N.D.
	F	53	Gingiva	N.D.	CR	Reduce IS	N.D.	+	N.D.	N.D.	N.D.
	F	73	Gingiva	N.D.	CR	Reduce IS	N.D.	+	N.D.	N.D.	N.D.
	M	51	Gingiva	Age	CR	R-CHOP	N.D.	+	N.D.	N.D.	N.D.
	F	65	Gingiva	N.D.	CR	Reduce IS	N.D.	+	N.D.	N.D.	N.D.
	F	71	Tongue	N.D.	N.D.	Reduce IS	N.D.	+	N.D.	N.D.	N.D.
Hujoel et al. 2018 [17]	F	75	Palate, Maxillary gingiva	IS (AZA for Crohn's disease)	CR	Reduce IS	N.D.	+	−	N.D.	N.D.
McCormack et al. 2018 [18]	M	79	Maxillary gingiva	Age	N.D.	N.D.	N.D.	+	+	N.D.	−
Pina-Oviedo et al. 2018 [19]	F	82	Tongue	Bendamustine and FCR for CLL	D (unknown)	N.D.	N.D.	+	+	+	+
Ravi et al. 2018 [20]	F	59	Buccal mucosa	IS (MTX for RA therapy)	CR	Reduce IS	N.D.	+	+	+	+
Prieto-Torres et al. 2019 [14]	M	74	Palate, Tongue	IS (MTX for RA therapy)	CR	Reduce IS	N.D.	+	+	N.D.	+
	F	75	Mandibular gingiva	IS (MTX for RA therapy)	CR	Reduce IS	N.D.	+	+	N.D.	N.D.
	F	87	Floor of mouth	Age	CR	RT	N.D.	+	−	N.D.	−
Satou et al. 2019 [15]	F	52	Gingiva	IS (MTX for RA therapy)	CR	Reduce IS, ABVD	+	+	+	N.D.	N.D.
	F	77	Tongue	IS (MTX for RA therapy)	CR	Reduce IS	+	+	+	N.D.	N.D.
Daroontum et al. 2019 [16]	M	75	Mandibular gingiva	Age	CR	R-CHOP, RT for DLBCL	N.D.	+	+	N.D.	N.D.
Ikeda et al. 2020 [11]	M	91	Gingiva	IS (MTX for RA therapy)	N.D.	R-THPCOP	N.D.	+	N.D.	N.D.	N.D.
	M	80	Gingiva	Age	N.D.	N.D.	N.D.	N.D.	-	N.D.	N.D.
	M	69	Gingiva	Age	N.D.	R-CHOP	N.D.	N.D.	-	N.D.	N.D.
	M	81	Oral mucosa	IS (MTX for RA therapy)	CR	Reduce IS, THPCOP	N.D.	+	-	N.D.	N.D.
	M	79	Angle of mouth	IS(HU for polycythemia therapy)	PR	Reduce IS	N.D.	+	-	N.D.	N.D.
	F	73	Oral mucosa	IS (MTX for RA therapy)	CR	Reduce IS	N.D.	+	-	N.D.	N.D.
	F	66	Tongue	IS (MTX for RA therapy)	PR	Reduce IS	N.D.	+	-	N.D.	N.D.
	F	70	Gingiva	IS (MTX for RA therapy)	CR	Reduce IS	N.D.	+	-	N.D.	N.D.
	F	76	Gingiva	IS (MTX for RA therapy)	CR	Reduce IS	N.D.	+	-	N.D.	N.D.
	F	78	Gingiva	IS (MTX for RA therapy)	PR	Reduce IS	N.D.	+	-	N.D.	N.D.
	F	75	Tongue	IS (MTX for RA therapy)	PR	Reduce IS	N.D.	+	-	N.D.	N.D.
	M	78	Oral mucosa	IS (MTX for RA therapy)	PR	Reduce IS	N.D.	+	-	N.D.	N.D.
	F	85	Gingiva	IS (MTX for RA therapy)	CR	Reduce IS	N.D.	+	-	N.D.	N.D.
	F	71	Gingiva	IS (MTX for RA therapy)	N.D.	Reduce IS	N.D.	+	-	N.D.	N.D.
	M	67	Gingiva	IS (MTX for RA therapy)	CR	Reduce IS	N.D.	+	-	N.D.	N.D.
	F	65	Gingiva	IS (MTX for RA therapy)	CR	Reduce IS	N.D.	+	-	N.D.	N.D.
	M	54	Buccal mucosa	IS (MTX for RA therapy)	PR	Reduce IS	N.D.	+	-	N.D.	N.D.
	F	67	Gingiva	IS (MTX for RA therapy)	CR	Reduce IS	N.D.	+	-	N.D.	N.D.
	M	75	Gingiva	IS (MTX for RA therapy)	CR	Reduce IS	N.D.	+	-	N.D.	N.D.
	F	86	Gingiva	IS (MTX for RA therapy)	CR	Reduce IS	N.D.	+	-	N.D.	N.D.
Li et al. 2020 [12]	M	59	Mandibular gingiva	IS(pemphigus vulgaris forPSL and MPA）	CR	Reduce IS, Surgery	N.D.	+	+	N.D.	N.D.
Shiraiwa et al. 2020 [13]	M	72	Tongue	IS (MTX and PSL for RA therapy)	CR	Reduce IS	N.D.	+	N.D.	N.D.	N.D.
	F	79	Gingiva	IS (MTX for RA therapy)	CR	Reduce IS	N.D.	+	N.D.	N.D.	N.D.
	M	61	Tongue	IS (MTX and PSL and SASP for RA therapy)	CR	Reduce IS	N.D.	+	N.D.	N.D.	N.D.
	M	67	Oral mucosa	IS (MTX and PSL for RA therapy)	CR	Reduce IS	N.D.	+	N.D.	N.D.	N.D.
	M	70	Gingiva	IS (MTX for RA therapy)	CR	Reduce IS	N.D.	+	N.D.	N.D.	N.D.
Obata et al. 2021 [8]	M	75	Mandibular gingiva	IS (MTX and PSL for RA therapy)	CR	Reduce IS	N.D.	+	+	+	N.D.
	M	80	Maxillary gingiva	IS (MTX for RA therapy)	CR	Reduce IS	N.D.	+	+	N.D.	N.D.
	F	58	Maxillary gingiva	IS (MTX and PSLfor RA therapy)	CR	Reduce IS	N.D.	+	+	N.D.	N.D.
	F	67	Maxillary gingiva	IS (MTX and PSL for RA therapy)	CR	Reduce IS	N.D.	+	+	N.D.	N.D.
	F	73	Maxillary gingiva	IS (MTX and TAC for RA therapy)	CR	Reduce IS	N.D.	+	+	N.D.	N.D.
	F	74	Mandibular gingiva	IS (MTX and TAC for RA and IP and RA therapy)	CR	Reduce IS	N.D.	+	+	N.D.	N.D.
	F	77	Mandibular gingiva	IS (MTX and BUC for RA therapy)	CR	Reduce IS	N.D.	+	+	N.D.	N.D.
	F	79	Mandibular gingiva	IS (MTX and PSLfor RA therapy)	CR	Reduce IS	N.D.	+	+	N.D.	N.D.
	F	81	Maxillary gingiva	IS (MTX and PSL for RA therapy)	CR	Reduce IS	N.D.	+	+	N.D.	N.D.
	F	87	Mandibular gingiva	IS (MTX for RA therapy)	CR	Reduce IS	N.D.	+	+	N.D.	N.D.
Xu et al. 2021 [9]	F	76	Buccal mucosa	N.D.	CR	Interferon α-1b	N.D.	+	+	+	N.D.
Fujimoto et al. 2021 [10]	M	77	Tongue	IS (PSL for DH therapy)	CR	Reduce IS	N.D.	+	+	N.D.	N.D.
	F	70	Lip	IS (PSL for RA therapy)	CR	Reduce IS	N.D.	+	+	N.D.	N.D.
	F	72	Mandibular gingiva	IS (MTX and TAC for RA therapy)	CR	Reduce IS	N.D.	+	+	N.D.	N.D.
Bott et al. 2022 [4]	M	89	Lip	CLL	D (unknown)	N.D.	N.D.	+	+	+	N.D.
Kunmongkolwut et al. 2022 [5]	F	52	Palate, Maxillary gingiva	IS (MTX and LMF for RA therapy)	CR	Reduce IS	N.D.	+	+	N.D.	N.D.
Kawamura et al. 2022 [6]	M	65	Retromolar region	IS (MTX for RA therapy)	N.D.	Reduce IS	N.D.	+	N.D.	N.D.	N.D.
Eleftheriadis et al. 2022 [7]	F	69	Lip	IS (PSL and MPA for kidney transplantation)	CR	Reduce IS, RIX, Surgery	N.D.	N.D.	N.D.	N.D.	N.D.
Sugisaki et al. 2024 [24]	F	80	Mandibular gingiva	MTX	CR	Reduce IS, Surgery	N.D.	+	N.D.	N.D.	+
Our case 2023	F	73	Maxillary gingiva	IS (MTX for RA therapy)	CR	Surgery	+	N.D.	+	+	+

**Figure 9:**
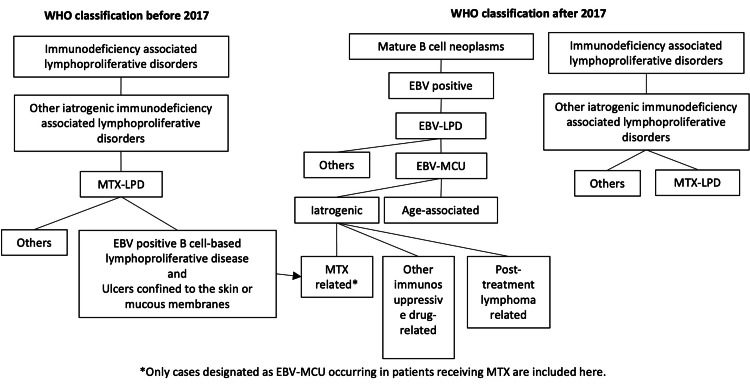
Positioning of EBV-MCU in the WHO 2017 classification MTX-LPD, methotrexate-related lymphoproliferative disorder; EBV-LPD, Epstein-Barr virus-positive lymphoproliferative disorder; EBV-MCU, EBV-positive mucocutaneous ulcer The diagram was drawn by the authors of this article.

The causes of disease are broadly classified into iatrogenic and age-associated. Iatrogenic causes are classified as (I) those caused by the use of immunosuppressive drugs such as MTX, azathioprine, mycophenolic acid, prednisolone, rituximab, and cyclosporine A, and (II) those caused by post-treatment lymphoma. Among cases of MTX-LPD diagnosed via the conventional classification, the condition with extranodal lesions and well-defined ulcerations confined to the skin, oral cavity, or gastrointestinal mucosa, accompanied by the proliferation of EBV-positive B lymphocytes, corresponds to the newly introduced classification of EBV-MCU (Figure 9). In our case, localized ulceration of the oral mucosa was also observed in a patient who had been using MTX for a long period. Among 79 previously reported cases, 70 cases were classified according to the cause of occurrence: (I) 49 cases (70.0%) involved the use of MTX, prednisolone, or other immunosuppressive drugs for rheumatoid arthritis, dermatitis herpetiformis, post-renal transplantation, pemphigus vulgaris, or Crohn's disease, and 9 cases (12.9%) involved an immunosuppressive state, although no detailed disease description was given; and (II) 2 cases (2.9%) occurred in association with lymphoma. Ten cases were considered to have been caused by aging. EBV activation in patients with RA is thought to be due to the activation of EBV by immunosuppression with MTX, in addition to the activation of B cells and immune abnormalities caused by autoantigen stimulation. The oral mucosa is frequently affected by mechanical irritation from dentures and inflammation by oral bacteria, which can cause local mucosal damage. This is thought to reduce mucosal resistance and lead to the local proliferation of EBV-infected cells.

The histopathological features of EBV-MCU on HE staining include ulceration with a pleomorphic infiltrate composed of numerous transformed EBV-positive large, atypical lymphocytes, immunoblasts, and Reed-Sternberg-like cells [2]. A definitive diagnosis requires the presence of EBV-positive cells by EBV-encoded small RNA in situ hybridization (EBER-ISH) or LMP-1, which is specific for EBV-positive cells, and confirmation of the use of immunosuppressive drugs. Histopathology may reveal lymphocytic infiltrates of varying sizes, diffuse large B-cell lymphoma (DLBCL)-like CD20-positive cells, or Hodgkin/Reed-Sternberg cell-like cells. Therefore, it is important to discriminate EBV-MCU from DLBCL or Hodgkin’s lymphoma (HL). Among the 79 cases we followed, there was a tendency for more cases to be positive for CD20. The Ki-67 index was high, ranging from 60% to 95% in all previous cases. In the present case, LMP-1 was positive on immunostaining, the pan-B cell markers CD20 and PAX5 were positive, and the Ki-67 index was 80%, showing a histology similar to that of EBV-positive DLBCL. In addition to these histopathological and clinical findings, localized ulceration in the oral cavity led to the diagnosis of EBV-MCU. EBV-MCU is the first condition to be considered for differentiation, as it progresses more slowly than other EBV-LPDs and is often in remission after withdrawal of the drugs used [1].

Treatment with EBV-MCU is recommended as conservative therapy with follow-up after withdrawal or reduction of the causative drug. If remission is not achieved, aggressive treatment may be required, including CD20/CD30 antibody therapy, radiation therapy, local resection, and systemic chemotherapy, alone or in combination [3,25]. Of the 79 cases mentioned above, treatment with iatrogenic EBV-MCU and the prognosis were described in 46 cases. Forty cases (87.0%) were in remission with drug withdrawal alone, and 4 cases (8.6%) were in remission with drug withdrawal, drug therapy, or surgical resection. All cases were in complete remission without any recurrence. Although guidelines for the treatment of EBV-MCU have not yet been established, in a review by Roberts et al. [3], it was reported that if remission of the lesion is not achieved, aggressive treatment, such as local excision, may induce remission. In addition, no clear international guidelines have been established for the treatment of MTX-LPD, which was classified before EBV-MCU was established as a subclass of Mature B cell neoplasms, but the latest Japanese guidelines [25] state that about 2/3 of patients will recover after discontinuation of the drug, but if the disease does not resolve with discontinuation of immunosuppressive therapy alone, consultation with the relevant department and biopsy should be aggressively considered. In the present case, based on the above, healing was achieved by surgical resection of the lesion and extraction of the non-preservable teeth without drug withdrawal. Teeth with significant mobility that cannot be preserved are thought to cause local mucosal damage owing to intraoral bacterial inflammation. Therefore, when immunosuppressive drugs cannot be withdrawn, as in our case, tooth extraction is considered appropriate for improving the oral environment.

EBV-MCU is a new category in the 2017 WHO classification; however, as mentioned above, many patients achieve remission with drug withdrawal or dose reduction, so it is necessary to confirm whether or not immunosuppressive drugs have been used. Therefore, it is necessary to correctly diagnose this disease and avoid excessive treatment. Surgical treatment when the offending agent cannot be discontinued does not always result in remission and may even worsen the lesion. Therefore, in such cases, a treatment decision should be made after a thorough discussion with the rheumatologist, hematologist/oncologist, and patient regarding non-surgical treatment options. Meanwhile, improvement of the oral environment through an excisional biopsy and tooth extraction, as in the present case, may lead to a cure.

## Conclusions

We encountered a case of EBV-MCU in the maxillary gingiva that went into remission after local environmental remediation due to difficulty in MTX withdrawal. This case report and literature review provide important insights into the clinical presentation, diagnosis, and treatment of EBV-MCU and may assist clinicians in its appropriate management.
